# Prostaglandin E2 potentiation of P2X3 receptor mediated currents in dorsal root ganglion neurons

**DOI:** 10.1186/1744-8069-3-22

**Published:** 2007-08-10

**Authors:** Congying Wang, Guang-Wen Li, Li-Yen Mae Huang

**Affiliations:** 1 Department of Neuroscience and Cell Biology, University of Texas Medical Branch, Galveston, TX 77555-1069, USA

## Abstract

Prostaglandin E2 (PGE2) is a well-known inflammatory mediator that enhances the excitability of DRG neurons. Homomeric P2X3 and heteromeric P2X2/3 receptors are abundantly expressed in dorsal root ganglia (DRG) neurons and participate in the transmission of nociceptive signals. The interaction between PGE2 and P2X3 receptors has not been well delineated. We studied the actions of PGE2 on ATP-activated currents in dissociated DRG neurons under voltage-clamp conditions. PGE2 had no effects on P2X2/3 receptor-mediated responses, but significantly potentiated fast-inactivating ATP currents mediated by homomeric P2X3 receptors. PGE2 exerted its action by activating EP3 receptors. To study the mechanism underlying the action of PGE2, we found that the adenylyl cyclase activator, forskolin and the membrane-permeable cAMP analogue, 8-Br-cAMP increased ATP currents, mimicking the effect of PGE2. In addition, forskolin occluded the enhancement produced by PGE2. The protein kinase A (PKA) inhibitors, H89 and PKA-I blocked the PGE2 effect. In contrast, the PKC inhibitor, bisindolymaleimide (Bis) did not change the potentiating action of PGE2. We further showed that PGE2 enhanced α,β-meATP-induced allodynia and hyperalgesia and the enhancement was blocked by H89. These observations suggest that PGE2 binds to EP3 receptors, resulting in the activation of cAMP/PKA signaling pathway and leading to an enhancement of P2X3 homomeric receptor-mediated ATP responses in DRG neurons.

## Background

ATP plays a prominent role in nociception. Its application onto human skin elicits pain [1,2]. Injection of ATP into the rat hindpaw reduces paw withdrawal latencies and produces flinching and writhing behaviors [3-6]. Recent studies of purinergic receptors in primary sensory dorsal root ganglion (DRG) neurons demonstrate that ATP gives rise to nociception by activating P2X receptors in primary sensory DRG neurons [7-9]. *In situ* hybridization assay indicates that P2X2-P2X6 mRNAs are present in DRG neurons [10]. P2X2 and P2X3 receptors are the major receptor types selectively expressed in peripheral and central terminals and the somata of DRG neurons [11-14]. These neurons are small (diameter d < 25 μm) and medium (25 < d < 40 μm) in size, bind isolectin B4 and express vanilloid TRPV1 receptors [15-18]. The importance of P2X3-containing receptors in nociception is further confirmed by the findings that nociceptive behaviors become greatly diminished in P2X3 knock-out mice [19,20] and in animals treated with P2X3 antisense oligonuclotides [21], small interfering RNA (siRNA) [22] or the specific P2X3 antagonist, A-37491 [23]. Electrophysiological studies indicate that ATP produces large inward currents by activating P2X3 homomeric and P2X2/3 heteromeric receptors, thus evoking depolarization in small and medium DRG neurons [11,13,14,24,25]. In the spinal dorsal horn, ATP, released from the central terminals of DRG neurons, acts on presynaptic P2X receptors to promote AMPA receptor-mediated synaptic transmission in nociceptive pathways [26-30]

An important characteristic of P2X3 receptor-mediated responses is its sensitivity to tissue and nerve injury. Nociceptive behaviors produced by ATP become greatly enhanced after inflammation [24] and nerve injury [31-33]. An increase in P2X3 receptor-mediated responses is one of the major reasons for the enhancement [11,31]. We found that the ATP currents in DRG neurons isolated from rats with inflammation or nerve injury are 2–3 fold larger [11,31]. The mechanisms responsible for the increase include upregulation of P2X3 receptors [11], enhancement of trafficking of P2X3 receptors toward the membrane [31] and activation of calmodulin protein kinase II [34]. The chemical mediator prostaglandin E2 (PGE2) is released during inflammation and sensitizes peripheral terminals of DRG neurons [35,36]. It increases capsaicin-evoked currents [37,38], promotes the release of substance P and CGRP from sensory neurons [39,40]. In the spinal cord, PGE2 dis-inhibits dorsal horn neurons by blocking inhibitory glycinergic synaptic responses [41,42]. PGE2 was found to enhance α,β-elicited nociceptive behaviors in rats [3]. The interaction between PGE2 and purinergic receptors has not been thoroughly investigated. Studying the action of PGE2 on ATP currents in DRG neurons, we found that PGE2 increases P2X3-receptor mediated ATP currents. Protein kinase A (PKA) mediates the potentiating action of PGE2.

## Results

### PGE2 potentiates P2X3 receptor-mediated ATP responses

**Figure 1:**
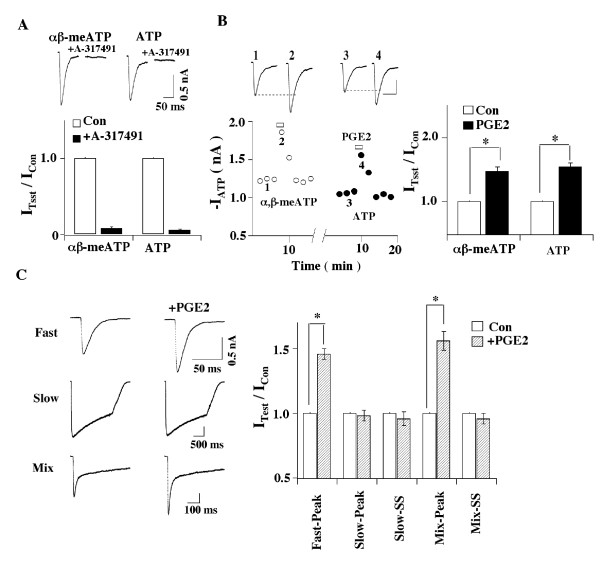
**Enhancement of fast-inactivating ATP currents by PGE2**. (A) PGE enhances P2X3 receptor-mediated ATP currents. α,β-meATP and ATP evoked similar fast-inactivating currents. Both currents were blocked by the specific P2X3 antagonist, A-317491. (B) In the same cell, PGE2 potentiated α,β-meATP- and ATP-evoked currents similarly. I_αβ-meATP _(PGE2)/I_αβ-meATP _= 1.47 ± 0.07, I_ATP _(PGE2)/I_ATP _= 1.54 ± 0.06 (* P < 0.05, n = 8). (C) Homomeric P2X3 receptors mediate the action of PGE2. (Left) A 2 sec ATP (10 μM) pulse elicited fast-inactivating (Fast), slow-inactivating (Slow) and mixed ATP currents in normal cells. Membrane was held at -60 mV. The currents were obtained from three different cells. (Right) After a 2-min application of PGE2 (1 μM), the fast ATP current was potentiated but the slow ATP current did not change. For the cell exhibiting mixed responses, PGE2 enhanced the peak, but not the steady state ATP currents. Bar graphs show the pooled data. PGE2 increased the peak amplitude of both fast-inactivating ATP currents [I_ATP _(PGE2)/I_ATP _= 1.46 ± 0.04 (n = 72)] and mixed ATP currents [I_ATP _(PGE2)/I_ATP _= 1.56 ± 0.08 (n = 6)] (* P < 0.05), which were mediated by homomeric P2X3 receptors. On the other hand, PGE2 had no effect on P2X2/3 receptor-mediated slow currents.

We first determined the effects of PGE2 on ATP or its analog, α,β-meATP-activated currents in DRG neurons. Only small (cross sectional area < 600 μm^2^) and medium (600–1200 μm^2^) cells, which are known to mediate the transmission of nociceptive signals [43], were used for the study. Application of 10 μM ATP or α,β-meATP at a holding potential of -60 mV elicited large inward currents in more than 80% of the neurons tested (Fig. 1A). The fast ATP or α, β-meATP-evoked currents are mediated by P2X3 receptors because α,β-meATP activates only P2X1 and P2X3 receptors [7] and the antagonist, A-317491, which acts specifically on P2X3 receptors [23], abolished both current responses (Fig. 1A). PGE2 (up to 100 μM), by itself, did not evoke any membrane currents (n = 6, data not shown). However, PGE2 (1 μM, 2 min application) enhanced the peak amplitude of both fast-inactivating ATP- and α,β-meATP-evoked currents (Fig. 1B). These results suggest that PGE2 potentiates homomeric P2X3 receptor-mediated responses.

To further validate this conclusion, the effects of PGE2 on P2X3 receptor-mediated currents were studied in detail. As described in our previous study [11], homomeric P2X3 and heteromeric P2X2/3 receptors are the major P2X receptors expressed in DRG neurons. The current responses can be categorized based on their kinetics of inactivation as fast, slow, and mixed responses. Fast-inactivating currents are mediated by homomeric P2X3 receptors, slow-inactivating currents by heteromeric P2X2/3 receptors and mixed currents by both homomeric P2X3 and heteromeric P2X2/3 receptors [11,13]. We found that PGE2 (1 μM) potentiated the peak amplitude of fast-inactivating ATP currents in 63% (72 out of 114) of the neurons tested (Fig. 1C). PGE2 had no effects on fast ATP currents in 22% of cells and reduced fast ATP responses in the rest 15% of cells (I_ATP _(with PGE2)/I_ATP _= 0.57 ± 0.08, n = 17) (data not shown). Only the potentiating effects of PGE2 on fast-inactivating ATP currents were investigated in detail. PGE2 did not alter slow-inactivating ATP currents (Fig. 1C). For mixed ATP responses, PGE2 increased the peak amplitude of the currents (I_ATP _(with PGE2)/I_ATP _= 1.56 ± 0.08, n = 6), while it had no effect on the steady-state currents (Fig. 1C). These results suggest that PGE2 acts on the homomeric P2X3 receptors, but not on the heteromeric P2X2/3 receptors.

### The characteristics of PGE2 actions on fast-inactivating ATP currents

**Figure 2:**
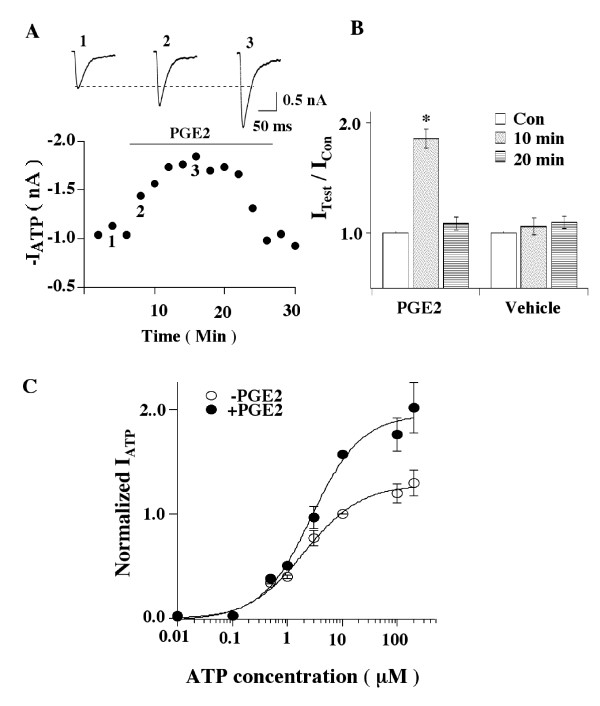
**Characteristics of PGE2 actions**. (A) Time course of PGE2 effects on fast ATP currents. ATP pulses were applied to the recorded cell every 2 min. After ATP responses reached a steady state, 1 μM PGE2 was applied to the bath. The fast ATP response was increased by 48% [I_ATP _(with PGE2)/I_ATP _= 1.48 ± 0.07, n = 6] two min later and the increase reached a peak level [I_ATP _(with PGE2)/I_ATP _= 1.90 ± 0.11] 10 min after PGE2 application. Numbers in the graph correspond to the original traces shown above. (B) Bar graphs are the pooled data from 5 cells (* P < 0.05). (C) PGE2 does not change the affinity of ATP for P2X3 receptors. Fast ATP currents were measured at different ATP concentrations in control and in 1 μM PGE2. For the purpose of normalization among cells, the responses to 10 μM ATP in control solution in all of the cells were measured. Each data point was obtained from studies of 4–6 cells. The data points at 0.01 and 0.1 μM ATP for control and PGE2 groups overlapped. The dose-response curves were fit by the Hill equation with Hill coefficient = 1. For control, EC_50 _= 2.02 ± 0.4 μM. For PGE2, EC_50 _= 2.77 ± 0.58 μM. PGE2 treatment does not alter ATP affinities for P2X receptors, although it greatly enhances peak ATP responses.

The time course of PGE2 (1 μM) effects was then studied (Fig. 2A). ATP (10 μM) was repeatedly applied to the tested cell every two min, a period that allowed P2X3 receptors to recover from inactivation sufficiently to give consistent current responses [11,44]. Two minutes after PGE2, fast ATP currents were increased by 48%, reaching a saturating value 10 min later. The enhancing effect was maintained at the peak level for another 4–6 min and then started to decay. Thus, the action of PGE2 on the fast-inactivating ATP currents desensitizes. Vehicle (0.1% DMSO) had no effect on the fast ATP currents (Fig. 2B). We chose to measure currents following a brief (2 min) application of PGE2 (1 μM) in most experiments because the application period allowed the potentiating effects of PGE2 to reach a sufficiently high level and to dissipate within 4 min.

The PGE2 effects on the kinetics of ATP currents were examined. The activation time (T_a_) of the fast current response was calculated by measuring the duration between 10 and 90% of the peak value. The inactivation time constants (τ_1in _and τ_2in_) were obtained by fitting the inactivating phase of the current with a sum of two exponentials. The changes in T_a_, τ_1in _and τ_2in _were not significantly different after PGE2 treatment (Table 1), suggesting that PGE2 does not alter the kinetics of the fast-inactivating ATP currents.

**Table 1:** Kinetics of ATP-evoked fast-inactivating currents in control (Con) and PGE2 treated DRG neurons.

	T_a_	τ_1in_	τ_2in_
Con	5.9 ± 1.1	38.1 ± 6.6	377.3 ± 95.2
PGE2	5.7 ± 0.9	46.1 ± 7.8	432.3 ± 121.2

Ta: Activation time, i.e., duration between 10 and 90% of peak current values.

τ_1in _and τ_2in_: Inactivation time constants. Data represent the mean ± SEM; n = 15. The differences in T_a_, T_1in _and T_2in _in Con and PGE2 neurons were not significant.

We then determined whether PGE2 changes the EC_50 _of ATP for P2X3 receptors. Dose-responses of ATP responses in the absence and presence of PGE2 (1 μM) were examined (Fig. 2C). ATP, at 200 μM, elicited maximal ATP currents in both cases, although the level of maximal response in PGE2 was 1.55-fold larger. Dose-response curves were fit with the Hill equation. The EC_50 _was 2.02 ± 0.4 μM in control and 2.77 ± 0.58 μM in PGE2 treated cells. Thus, the EC_50 _of ATP for P2X3 receptors was not significantly changed by PGE2. We conclude that the potentiation of ATP responses by PGE2 is not due to a change in the apparent affinity of ATP for P2X3 receptors.

### EP3 receptors mediate the potentiating effect of PGE2

**Figure 3:**
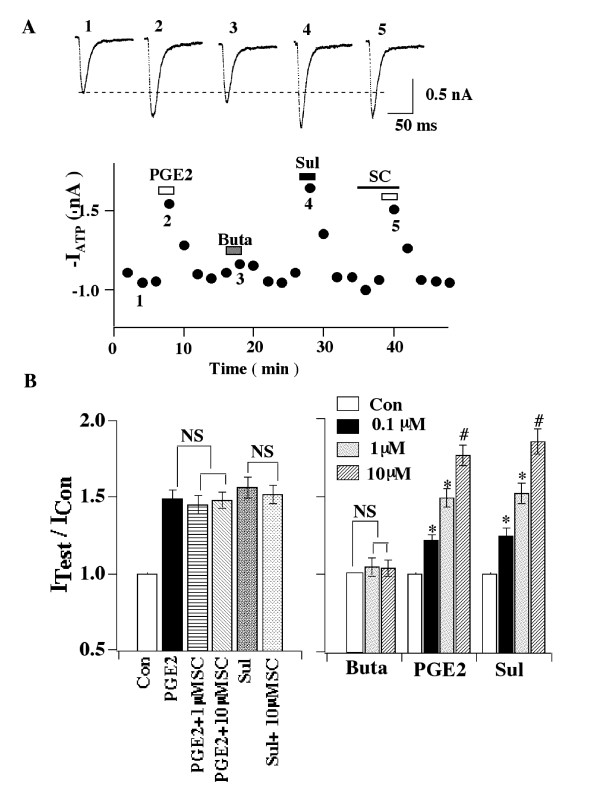
**EP3 receptors mediate the potentiation of ATP responses by PGE2**. (A) In the cell shown, PGE2 increased ATP currents. However, the EP2 receptor-specific agonist, butaprost (Buta) (1 μM), had no effect on the ATP responses. The EP3/EP1 agonist, sulprostone (Sul) (1 μM) mimicked the enhancing effect of PGE2. The EP1 antagonist, SC-19220 (SC) (1 μM), could not block the PGE2 potentiating effect. (B) Bar graphs are the pooled data from 3–8 cells. Only Sul mimicked the PGE2 effect on ATP currents. PGE2 effects: 0.1 μM PGE2, I_ATP _(PGE2)/I_ATP _= 1.22 ± 0.04; 1 μM PGE2, I_ATP _(PGE2)/I_ATP_= 1.49 ± 0.06; 10 μM PGE2, I_ATP _(PGE2)/I_ATP _= 1.76 ± 0.07. Sul effects: 0.1 μM Sul, I_ATP _ (Sul)/I_ATP_ = 1.25 ± 0.06; 1 μM Sul, I_ATP _(Sul)/I_ATP_ = 1.52 ± 0.07; 10 μM Sul, I_ATP _(Sul)/I_ATP _= 1.85 ± 0.08. SC could not inhibit PGE2- or Sul-elicited potentiation. The results suggest that the PGE2 effect is mediated by EP3 receptors. (* P < 0.05, # P < 0.01, NS = not significant)

The prostanoid receptors (EP1-EP4) that mediate the potentiating effects of PGE2 (Fig. 3) were determined. The selective EP1 antagonist, SC-19220 [45], at 1 or 10 μM, could not block the enhancing effects of PGE2, suggesting that EP1 is not likely to be involved. Because no specific antagonists for EP2 and EP3 were readily available, agonists (butaporst for EP2 and sulprostone for EP3) were used. The selective EP2 agonist, butaporst [46], at either 1 or 10 μM, did not affect ATP currents. In contrast, the EP3 agonist, sulprostone, at 0.1, 1, 10 μM, progressively potentiated ATP responses, mimicking the effect of PGE2. Although sulprostone also has a low affinity for EP1 receptors [45,46], the inability of the EP1 antagonist, SC-19220 on sulprostone action has led us to conclude that EP3 receptors mediate the effects of PGE2. Due to the lack of proper agonists and antagonists, the involvement of EP4 receptors was not studied.

### PGE2 potentiation is mediated by PKA

**Figure 4:**
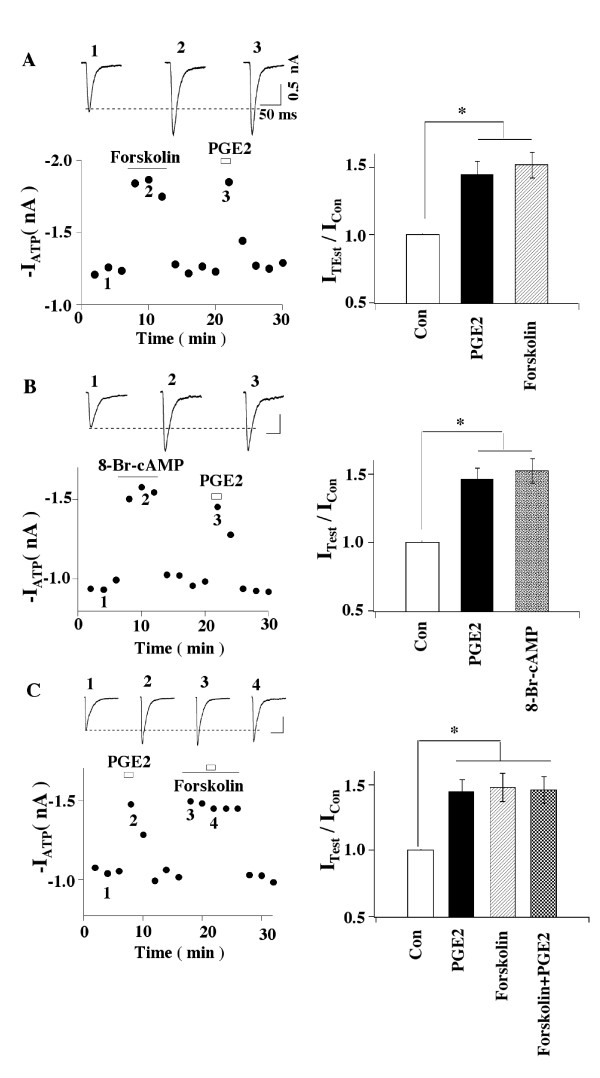
**Forskolin and 8-Br-cAMP mimic PGE2-elicited increase in ATP currents**. (A) Effects of forskolin. (Left) Examples of the action of forskolin (1 μM, 6 min) or PGE2 (1 μM, 2 min) on fast-inactivating ATP currents in the same cell. (Right) Forskolin and PGE2 potentiatied ATP currents (I_ATP _(PGE2)/I_ATP _= 1.45 ± 0.09; I_ATP _(Forskolin)/I_ATP _= 1.52 ± 0.10, n = 5). (B) 8-Br-cAMP enhanced ATP currents (I_ATP _(PGE2)/I_ATP _= 1.46 ± 0.08, I_ATP_(8-Br-cAMP)/IATP = 1.55 ± 0.09, n = 4) (*P < 0.05). (C) Forskolin occludes the effect of PGE2. In the presence of foskolin, PGE2 could not further increase ATP currents.

**Figure 5:**
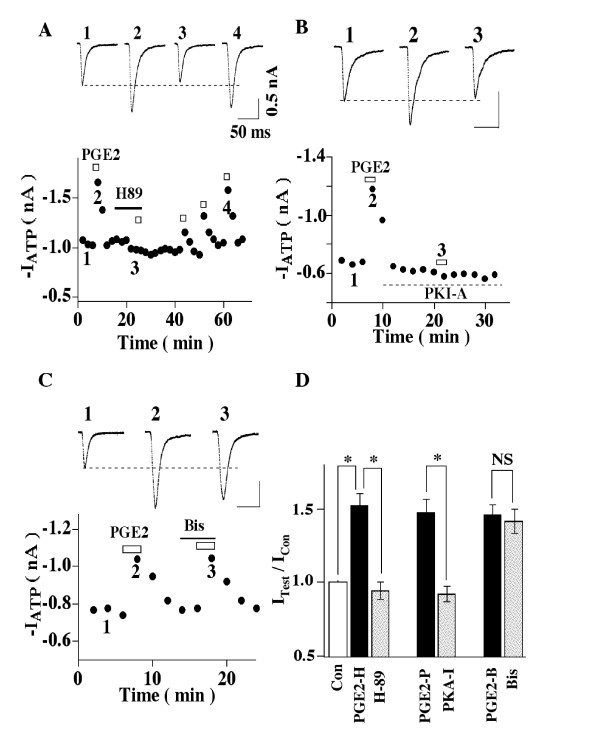
**PKA mediates PGE2-induced increases in ATP responses**. (A) Effects of the PKA inhibitor, H89. Treatment with H89 (1 μM) reduced ATP currents moderately. In the presence of H89, PGE2 could no longer increase ATP currents. The block of H89 was reversed rather slowly. After washout of H89 for 35 min, the PGE2 potentiating effect returned to the control levels. (B) Effects of the specific membrane impermeant inhibitor PKA-inhibitor (6–22) (PKA-I). PKA-I (0.2 μM) was included in the patch pipette. As the result of small diameter tip pipettes used in these experiments, PKA-I did not reach its final concentration in the cell interior several minutes after whole cell recording was established. This allowed us to obtain a few control responses to PGE2 before PKA-I became effective. With PKA-I, PGE2 no longer potentiated ATP responses. (C) Effects of the PKC inhibitor, Bis. Pretreatment of the cell with Bis (1 μM) did not affect the potentiating effect of PGE2. (D) Average effects of PGE2, (PGE2+ H89), (PGE2+PKA-I) and (PGE2+Bis). For each protein kinase inhibitor, the effects of PGE2 and (PGE2+ inhibitor) were examined in the same cell. For H89, I_ATP _(PGE2-H)/I_ATP _= 1.52 ± 0.08; I_ATP _(PGE2-H+H89)/I_ATP _= 0.94 ± 0.06. For PKA-I, (I_ATP _(PGE2-P)/I_ATP_ = 1.48 ± 0.09; I_ATP _(PGE2-P+PKA-I)/I_ATP _= 0.92 ± 0.05. For Bis, I_ATP _(PGE2-B)/I_ATP _= 1.46 ± 0.07, I_ATP _(PGE2-B+Bis)/I_ATP_ = 1.42 ± 0.08. Five cells were tested in each group (* P < 0.05, NS = not significant).

Since PKA is known to participate in the actions of PGE2, its role in the potentiation of ATP responses was studied. We first studied that action of the adenylyl cyclase activator, forskolin and a membrane-permeable cAMP analogue, 8-Br-cAMP on P2X3-receptor mediated currents. Both forskolin and 8-Br-cAMP increased ATP currents, mimicking the action of PGE2 (Figs 4A and 4B). To make sure that PGE2 used the same signaling pathway as forskolin, an occlusion experiment was performed. After forskolin effect on ATP currents was established, PGE2 was then applied to the cell (Fig. 4C). PGE2 no long could increase ATP currents in the presence of forskolin. The results suggest an involvement of cAMP/PKA. We then studied the effect of a membrane-permeant PKA inhibitor, H89, on the enhancing effect of PGE2. We first made sure that the tested cell responded to PGE2 and then perfused H89 (1 μM) onto the recorded cell (Fig. 5A). In the presence of H89 (1 μM), ATP responses declined somewhat. After 10 min pretreatment with H89, PGE2 no longer could enhance ATP currents (Figs. 5A and 5D), suggesting that PKA is involved in the modulatory action of PGE2. To confirm the conclusion, we studied the effect of a specific membrane impermeant PKA inhibitor, PKA-I, on the PGE2 action (Fig. 5B). PKA-I was perfused into the cell through a patch electrode. In order to measure PGE2 potentiation before PKA-I reached the cell interior, patch electrodes of small tips (electrode resistance = 7–8 Mohm) were used to slow the intracellular perfusion of PKA-I. In the presence of PKA-I, the potentiating effect of PGE2 was completely blocked (Figs. 5B, D), confirming that cAMP/PKA signaling is involved in the PGE2 enhancement of ATP currents. The role of PKC was also investigated. We found that bath-application of the membrane-permeant PKC inhibitor, Bis (1 μM), onto the recorded cell had no effect on the PGE2 action (Figs. 5C and 5D). Thus, PKC does not participate in the PGE2 modulation of ATP currents in normal DRG neurons.

### PGE2 enhances α,β-meATP induced hyperalgesia and allodynia

**Figure 6:**
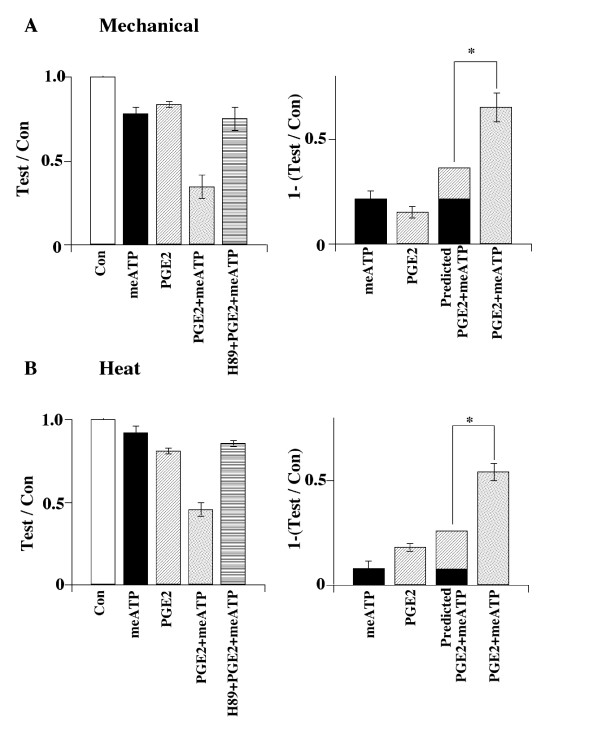
**PGE2 sensitizes the mechanical allodynia and thermal hyperalgesia produced by α,β-meATP**. (A) Mechanical allodynia. Paw withdrawal threshold was measured 10 min after paw injection of α,β-meATP (1 nmol/50 μl) or PGE2 ((0.05 nmol/50 μl). The data were normalized with baseline responses before either injection. α,β-meATP and PGE2 applied individually resulted in a moderate decrease in the threshold [(1-meATP/Con) = 0.22 ± 0.04; (1-PGE/Con) = 0.16 ± 0.03, n = 3)]. However, co-injection of α,β-meATP and PGE2 produced a much larger decrease in the threshold [1-(meATP+PGE)/Con = 0.65 ± 0.07, n = 3] than adding the threshold reduction produced by α,β-meATP and by PGE2. H89 (0.5 nmol/50 μl) reversed the enhanced allodynia produced by PGE2. (B) Thermal hyperalgesia. α,β-meATP and PGE2, applied separately, produced a small reduction in the paw withdrawal latency [(1-meATP/Con) = 0.08 ± 0.04; (1-PGE/Con) = 0.18 ± 0.02, n = 3]. Co-injection of PGE2 and α,β-meATP produced a much larger reduction in the paw withdrawal latency [(1-(meATP+PGE)/Con = 0.54 ± 0.04, n = 3]. H89 blocked the enhancing effect of PGE2. (* P < 0.05, Two-way ANOVA).

We also determined whether the PGE2 enhancing effect on P2X3 receptor-mediated responses could be observed behaviorally (Fig. 6). Low concentrations of α,β-meATP (1 nmol/50 μl) and PGE2 (0.05 nmol/50 μl) were used for the study. By keeping the individual effects of either α,β-meATP or PGE2 small, an enhancing effect of (α,β-meATP +PGE2), if it existed, could be easily distinguishable. Intraplantar injection of α,β-meATP or PGE2 lowered the paw withdrawal (PW) mechanical thresholds by 21.9 ± 3.8% and 15.5 ± 3.0% (n = 3) respectively (Fig. 6A). However, the PW threshold was lowered by 65.3 ± 6.9% (n = 3) when α,β-meATP and PGE2 were applied simultaneously. Thus, the combination of α,β-meATP and PGE2 produced a significantly stronger response than adding the responses produced by separate application of α,β-meATP and PGE2. Intraplantar pretreatment with the PKA inhibitor, H89 (0.5 nmol/50 μl), reversed the enhanced allodynia produced by PGE2 (Fig 6A). Similar experiments were conducted to study PGE2 effects on the PW latency in response to thermal stimulation (Fig. 6B). α,β-meATP + PGE2 reduced the PW latency significantly more than the combined reduction of PW latencies produced by separate application of α,β-meATP and PGE2. H89 again reversed the enhanced hyperalgesia produced by PGE2. These observations suggest that PGE2 sensitizes α,β-meATP-induced allodynia and hyperalgesia through a PKA signaling pathway.

## Discussion

We found that PGE2 increases the amplitude of P2X3 homomeric receptor-mediated ATP currents without changing the kinetics of the currents in a majority of small and medium DRG neurons (Fig. 1, Table 1). In contrast, PGE2 had no effect on P2X2/3 heteromeric receptors. The enhancement of ATP responses is not due to a change in the apparent affinity of ATP for P2X3 receptors (Fig. 2) but the result of activation of PKA by PGE2. The conclusion is supported by the observations: (1) the PKA activators, e.g., forskolin, not only mimics the action of PGE2 but occludes the effect of PGE2 (Fig. 4) and (2) the PKA inhibitors, H89 and PKA-I, block the action of PGE2 (Fig. 5). Our conclusion is further supported by the behavioral observations that PGE2 sensitizes the α,β-meATP-induced spontaneous flinching responses [3] and enhances α,β-meATP-induced allodynia and hyperalgesia (Fig. 6). We further showed that the enhancement could be blocked by intraplantar injection of H89 (Fig. 6). The molecular mechanism responsible for PKA-induced potentiation of ATP current is not well understood. Few studies were conducted to study the conserved PKA site on the P2X receptor molecules. There is evidence that phosphorylation at Ser431 in the C-terminus of P2X2 receptors reduces ATP currents mediated by P2X2 receptors transfected in HEK293 cells [47]. On the other hand, the adenylyl cyclase, forskolin, was found to increase ATP currents in HEK cells expressing P2X4 receptors although the phosphorylation site on the receptor has not been identified [48]. The factor contributing to the differential actions of PKA on different P2X receptor subtypes has yet to be determined. It is unclear how phosphorylation of the conserved PKA site changes P2X receptor activity. A number of possibilities have been proposed by studying phosphorylation of sites other than the PKA site. Phosphorylation of the PKC sites in P2X1 and P2X2 receptor slowed the rate of inactivation of ATP currents [49,50]. Calcium/calmodulin-dependent protein kinase II was found to enhance P2X3 receptor activity by promoting trafficking of the receptor to the membrane [34]. Phosphorylation of regulatory proteins associated with P2X1 or P2X7 receptor increased the activity of the P2X receptors [51,52].

To identify the EP receptor involved in the action of PGE2 is challenging because agonists and antagonists of EP receptors are not completely selective [53]. By using a combination of the agonists and antagonist, we conclude that EP3 mediates the potentiating action of PGE2. This conclusion is consistent with the study of Southall and Vasko [39] who used reverse transcription polymerase chain reaction and antisense to identify the EP receptors participate in PGE2-induced sensitization of DRG neurons. They found that although EP1, 2, 3C and 4 are expressed in DRG neurons, as observed by others [54], only EP3C and EP4 receptor subtypes mediate the PGE2-induced cAMP production and increased release of substance P and CGRP. We have not determined which EP3 splice variant participates in the potentiating action of ATP. Given that EP3A and EP3B mRNA are not found in DRGs [39] and EP3C (i.e., EP3γ) is the only EP3 variant coupled to Gs to produce cAMP [55], we suggest that PGE2 acts on EP3C to potentiate the response of P2X3 currents. In addition to increasing ATP currents, PGE2 was also found to decrease or have no effect on ATP currents in some DRG neurons. The reason for this variability has not been studied. DRG neurons expressing other EP receptor subtypes or devoid of EP receptor expression could contribute to the varying PGE2 actions. For example, the EP3D receptor is known to couple to Gi or Gq to reduce cAMP level in cells [45].

It is well documented that PGE2 produces sensitization of TRPV1 receptors [38] and TTX-resistant Na^+ ^channels [36,56] in DRG neurons. Here, we show that the P2X3 receptor is another target of PGE2. Since P2X3 receptors play an important role in pain processing [12,13] and their activation is sensitive to tissue and nerve injury [3,11], the modulation by PGE2 would provide a way for sensory neurons to specifically respond to tissue injury. Our study elucidates the mechanism of an acute action of PGE2 on ATP currents. P2X3 receptors have been shown to undergo profound changes in their expression and trafficking after inflammation and nerve injury [11,31,34]. The binding properties of EP receptors [57] and the signaling of PGE2 can change in inflamed tissues [58-60]. It is of great interest to determine how PGE2 modulates P2X3 receptor-mediated responses after inflammation or nerve injury. A good understanding of the plasticity of PGE2 action on P2X receptors under injurious conditions will help us use the downstream targets of PGE2 for designing analgesics. Such therapeutic agents should be more specific and produce less side-effects, thus providing an alternative to cyclooxygenase 2 (COX2) inhibitors which can have devastating risks for patients [61,62].

## Conclusion

We demonstrate that PGE2 potentiates P2X3 receptor-mediated ATP currents in DRG neurons and enhances α,β meATP-induced allodynia and hyperalgesia by binding to EP3 receptors to activate the cAMP/PKA signaling cascade. Thus, the P2X3 receptor represents a downstream target of PGE2 and is likely a useful therapeutic drug target for treating inflammatory and neuropathic pain.

## Methods

### Animals

All animal procedures were in accordance with the guidelines of the National Institutes of Health and the International Association for the Study of Pain and approved by the Institutional Animal Care and Use Committee at the University of Texas Medical Branch. Sprague Dawley rats were used in the study. Rats of 25–35 d of age were used in the electrophysiology study; older rats (8–10 weeks old) were used in behavioral experiments.

### Electrophysiology

ATP currents were recorded from acutely dissociated neurons isolated from L4-5 DRGs of 25–35 d old rats. DRG neurons isolated from young rats survived better. The characteristics of ATP currents in young and adult DRGs were indistinguishable. DRGs were excised from sodium pentobarbital (50 mg/kg, i.p.) anesthetized rats and put in an ice-cold, oxygenated dissecting solution, which contained (in mM): 135 NaCl, 5 KCl, 2 KH_2_PO_4_, 1.5 CaCl_2_, 6 MgCl_2_, 10 glucose, and 10 HEPES, pH 7.2 (osmolarity, 305 mOsm). After removal of the connective tissue, the ganglia were put into a dissecting solution containing collagenase IV (1.0–1.5 mg/ml; Boehringer Mannheim, Indianapolis, IN) and trypsin (1.0 mg/ml; Sigma, St. Louis, MO) and incubated for 1 hr at 34.5°C. Afterward, DRGs were taken out from the enzyme solution, washed and put into another dissecting solution containing DNAase (0.5 mg/ml; Sigma). Ganglia were then triturated with fire-polished glass pipettes and the dissociated cells were placed on acid-cleaned glass coverslips. The experiments were performed at room temperature two hours after plating. Cells were continuously superfused (0.5 ml/min) with an external solution [130 mM NaCl/5 mM KCl/2 mM KH_2_PO_4_/2.5 mM CaCl_2_/1 mM MgCl_2_/10 mM HEPES/10 mM glucose, pH 7.3 (osmolarity, 295–300 mosM)]. In order to obtain a fast solution exchange, ATP (Sigma) was applied through an electronic valve with a solution exchange rate of 0.2 ms. This exchange rate was fast enough so that it would not limit peak ATP responses. Under voltage-clamp conditions, the whole-cell patch recording technique was used for current recordings. Membrane potential was held at -60 mV. Unless indicated, patch-clamp electrodes had a resistance of 3–5 Mohm when filled with the pipette solution, which contained (in mM): 145 K gluconate, 10 NaCl, 10 HEPES, 10 Glucose, 5 BAPTA and 1 CaCl_2_, pH 7.25 adjusted with KOH (osmolarity = 290 mOsm). The currents were filtered at 2–5 kHz and sampled at 100 μs per point.

### Behavioral experiments

Mechanical allodynia was quantified by the responses to von Frey filament stimulation using the 50% threshold methods [63]. Individual rats were placed on a metal mesh platform and under a plastic dome. Animals were habituated to the testing environment for at least 20 min before an experiment. A series of calibrated von Frey filaments of increasing strengths will be applied to the midplantar surface until a paw withdrawal occurred. The 50% threshold (expressed in grams), which is defined as 10 (Xf+ k × d)/10000 where Xf = value of the final von Frey filament unit used (in log units); k = tabular value of the pattern of positive/negative response; d = mean difference (in log units) between von Frey hairs, was determined. A cutoff threshold was 15 g to avoid tissue damage. All behavioral studies were performed under blind conditions. Saline (PBS) or α,β-meATP was injected into the rat paw. The α,β-meATP effect on PW threshold was measured 10 min after the injection when the response reached a peak level. To study the effect of PGE2 on the α,β-meATP response, α,β-meATP and PGE2 were applied simultaneously to the rat paw. To study the protein kinase-dependence of PGE2, the PKA antagonist, H89 was applied 20 min prior to the (PGE2+α,β-meATP) application.

Thermal hyperalgesia was examined by measuring paw withdrawal latencies (PWLs) using the radiant heat method [64]. A lamp was placed under the plantar surface of the rat hindpaw and the time elapsed from the onset of radiant heat stimulation to the withdrawal of the paw was recorded. The heat intensity was adjusted to give a baseline latency of ≈10 s; a cutoff time of 30 s was set to prevent tissue damage. To obtain baseline PWLs, three measurements separated by a 5-min interval were made for each rat's hind paw and scores were averaged.

### Drugs

Bisindolylmaleimide I (Bis), H89 dihydrochloride (H89), protein kinase A inhibitor 6–22 amide (PKA-I), were purchased from Calbiochem (La Jolla, CA). ATP, α,β-meATP, 8-Bromoadenosine 3',5'-cyclic monophosphate (8-Br-cAMP), A-316491 and forskolin were from Sigma (St. Louis, MO) ; PGE2, sulprostone, butaprost and SC-1220 were from Cayman Chemical (Ann Arbor, MI). Except for PKA-I, ATP and α,β-meATP, all other compounds were prepared in DMSO as stocks. The final concentrations used in experiments at least 1000 times lower than stocks concentrations. Stock solutions were stored at -20°C and diluted immediately before use.

### Data analyses

All data are expressed as mean ± SEM. Differences between two means were analyzed with paired or unpaired Student's t-test. Rise time (Ta) and inactivation time constants were used to determine kinetic properties of ATP responses. Ta of ATP currents were obtained by measuring the activation time between 10 and 90% of the peak value. The time constants of current inactivation were obtained by fitting the decay phase of current with exponential functions using the Levenberg-Marquardt algorithm. Comparisons between multiple means were done with one-way analysis of variance (ANOVA) followed by Newman Keuls *post hoc *test. A P < 0.05 was considered significant.

## Competing interests

The author(s) declare that they have no competing interests.

## Authors' contributions

CW designed and performed the electrophysiological experiments and drafted the manuscript. GL carried out the behavioral experiments. LMH designed and coordinated the study and prepared the manuscript. All authors read and approved the final manuscript.
